# Possible Insulinotropic Action of Apolipoprotein A–I Through the ABCA1/Cdc42/cAMP/PKA Pathway in MIN6 Cells

**DOI:** 10.3389/fendo.2018.00645

**Published:** 2018-10-30

**Authors:** Koki Matsumura, Naoki Tamasawa, Makoto Daimon

**Affiliations:** ^1^Graduate School of Medicine and School of Medicine, Hirosaki University, Hirosaki, Japan; ^2^Aomori Rosai Hospital, Hachinohe, Japan

**Keywords:** ApoA-I, HDL, insulin secretion, ABCA-1, Cdc42, cyclic AMP, PKA, MIN6

## Abstract

**Aims/Introduction:** We studied the mechanisms for the possible insulinotropic action of apolipoprotein (Apo) A–I in mouse insulinoma (MIN6) cells.

**Materials and Methods:** The effects of ApoA-I on cAMP production and glucose-stimulated insulin secretion (GSIS), and the dose dependency (ApoA-I at 5, 10, 25, and 50 μg/ml) were determined using MIN6 cells. The effects of the small-interference ribonucleic acid (siRNA) of ATP-binding cassette transporter A1(ABCA1) and Cell division control protein 42 homolog (Cdc42) on the insulinotropic action of ApoA-I was studied, as well as mRNA and protein levels of ABCA1 and Cdc42. Then, the influence of cAMP inhibitor SQ22536, and the cAMP-dependent protein kinase inhibitor Rp-cAMPS on ApoA-I action were studied.

**Results:** Addition of ApoA-I produced cAMP and increased insulin secretion, dose-dependently in high glucose concentration (25 mmmol/l). and ABCA1 protein and Cdc42 mRNA and protein were also enhanced. Specific ABCA1 and Cdc42 siRNA significantly decreased the effects of ApoA-I on insulin secretion compared with negative controls. Manifestations of ABCA1 and Cdc42 mRNA and protein were less than that of the negative control group. Both cAMP inhibiror (SQ22536) and protein kinases inhibitor (Rp-cAMPS) strongly inhibited the effects of ApoA-I on insulin secretion.

**Conclusions:** We demonstrated that ApoA-I enhances glucose-stimulated insulin release in high glucose at least partially through the ABCA1/Cdc42/cAMP/ Protein kinase A (PKA) pathway.

## Introduction

A low plasma concentration of HDL-cholesterol (HDL-C) is considered to be a strong, statistically independent risk factor for cardiovascular disease (CVD) in large clinical intervention studies (1–4). However, recent studies have reported the possibility that HDL-C levels *per se* have essential limitations as an indicator of the anti-atherogenic effects of HDL *in vivo* (5, 6), and various HDL functions have been reported to have more predictive value for atherosclerosis (5).

One of the most studied functions of HDL coupled with Apolipoprotein A-I (HDL/ApoA-I) is cholesterol efflux, by which cholesterol is transported from peripheral tissues and macrophages to the liver for excretion (reverse cholesterol transport (RCT)4), preventing lipid accumulation in peripheral tissues and the development of atherosclerosis (3–5, 7, 8). Besides cholesterol efflux, HDL/ApoA-I has many other physiological functions, including anti-oxidative, anti-inflammatory, anti-apoptotic, and anti-thrombotic roles (9, 10). These functions of HDL/ApoA-I have been explained through its interaction with the ATP-binding cassette transporter A1 (ABCA1), an integral cell membrane protein, and are understood to contribute to the preservation of cell function.

In patients with type 2 diabetes, low plasma HDL-C is one of the clinical features of dyslipidemia, which is associated with hypertriglyceridemia and small-dense low-density lipoprotein (LDL) (11). Furthermore, a recent study has revealed that cholesterol efflux capacity is reduced in diabetes due to “dysfunctional HDL” metabolism (12).

Studies on HDL/ApoA-I in relation to glucose metabolism have suggested that HDL/ApoA-I has additional beneficial actions relevant to diabetes mellitus. For example, HDL/ApoA-I may be involved in maintaining normal β cell function, acting to inhibit β cell apoptosis and to promote β cell survival (9, 10, 13). Then, increased glucose uptake into skeletal muscle via activation of the AMPK signaling pathway has also been reported (14, 15). Furthermore, recent studies have demonstrated that ApoA-I or remnant HDL particles enhance insulin secretion from mouse insulinoma (MIN6) cells or primary islets in a glucose-dependent manner, although the underlying mechanism has not been defined (13, 16).

Research has shown that the interaction of ApoA-I with ABCA1 triggers signal transduction pathways to mediate post-translational ABCA1 activity or lipid transport activity (14, 15, 17). Thus, ABCA1 is considered to be involved in insulin secretion from pancreatic β cells. Cell division control protein 42 homolog (Cdc42) is member of Rho GTPase superfamily, and reported to be activated by ApoA-I (18). Then, Cdc42 signaling has been reported to be essential for the second phase of insulin secretion (19).

In this study, we confirmed insulinotripic action of ApoA-I and then investigated the possible mechanism underlying the insulinotropic action through ABCA1/Cdc42/cAMP/PKA pathway in MIN6 cells.

## Materials and methods

### Cell culture and glucose-stimulated insulin secretion

The MIN6 cells used were a gift of Prof. Miyazaki (Division of Stem Cell Regulation Research, Osaka University Graduate School of Medicine, Osaka Japan). Measurement of insulin secretion of MIN6 cells was performed according to the original report (20). Cells (ca. 5 × 10^5^/100 μl) were placed in 24- or 96-well plates (100–300 μl) and grown for 1 day in DMEM containing 25 mmol/l glucose and 10% FBS at 37°C with 5% CO_2_. The cells were then washed twice in DMEM containing 0.5 mmol/l glucose and 10% FBS and cultured in this medium for 1 day. The medium was replaced with 0.5 ml of 0.5, 5.5, or 25 mmol/l glucose DMEM containing 5% FBS every 1 h.

All culture supernatants were collected after each incubation, centrifuged briefly to remove cell debris, and stored at −20°C before being studied using an enzyme-linked immunosorbent assay (mouse insulin ELISA; Mercodia, Uppsala, Sweden). The cells were harvested and a cell lysate was prepared with a mammalian protein extraction reagent (M-PER; Thermo Fisher Scientific, Waltham, MA, USA) for determination of cell protein.

### Measurement of cAMP in MIN6 cells mediated by glucose and ApoA-I

cAMP levels mediated by glucose and ApoA-I were firstly quantified in MIN6 Cells. After 1 h incubation with each condition with glucose and ApoA-I, cAMP was extracted from the cell using M-PER with proteinase inhibiter. Then, cAMP levels in cell lysates were quantified (pmol/mg cell protein) by ELISA (No. 581001, Cayman Chemical) (21).

The effect of glucose (final concentration 0.5, 5.5, and 25 mmol/l) on cAMP production was determined in MIN6 cells cultured in the DMEM medium with 50 μg/ml of ApoA-I (apolipoprotein A-I from human plasma; Sigma-Aldrich, St. Louis, MO, USA). Then, the effect of ApoA-I (final concentration 10, 25, and 50 ug/ml) on cAMP production was determined in the medium with 25 mmol/l of glucose.

### The insulinotropic action of ApoA-I in MIN6 cells

The effect of ApoA-I on glucose-stimulated insulin secretion (GSIS) was determined in MIN6 cells. Insulin secretion was measured in the medium without and with ApoA-I (5, 10, 25, or 50 μg/ml). Insulin secretion due to ApoA-I addition and dose dependency were determined under the same conditions with MIN6 cells.

### Effects of small-interference RNA transfection on insulin secretion in MIN6 cells

Involvement of ABCA1 and Cdc42 to ApoA-I induced insulin secretion was specifically examined using each small-interference RNA (siRNA). The MIN6 cells were transfected with small-interference RNA (GeneSolution siRNA; QIAGEN, Venlo, The Netherlands) of ABCA1 (GS11303), Cell division control protein 42 homolog (Cdc42) (GS12540), and negative control in the absence or presence of ApoA-I (50 μg/ml) using TaqMan® MicroRNA Assays (Invitrogen, Grand Island, NY, USA) 13. We described Sequences of siRNA of ABCA1, Cdc42, and negative control we used were as follows:

ABCA1: Mm_Abca1_5 (Target sequence: AACCACTTTGATACTGAATTA).

Cdc42: Mn_Cdc42_4 Target sequence: TTAAATCAAACTAAAGATTAA.

NC: Target sequence: AATTCTCCGAACGTGTCACGT.

After 24-h incubation, insulin levels in the medium, as well as mRNA and protein levels of ABCA1 and Cdc42, were determined with RT-PCR and western blotting, as detailed below.

### Quantitative RT-PCR for ABCA1 and Cdc42

Total RNA was extracted from MIN6 cells using an RNeasy Mini Kit (QIAGEN). The mRNAs for ABCA1, Cdc42, and Glyceraldehyde 3-phosphate dehydrogenase (GAPDH) were analyzed by quantitative RT-PCR (ABI PRISM 7700 Sequence Detection System; Applied Biosystems, Foster City, CA, USA). The TaqMan probes for ABCA1 (Mm00442646_m1), Cdc42 (Mm01194005_g1), and GAPDH (Mm99999915_g1) were also purchased from Applied Biosystems.

### Western blotting for ABCA1 and Cdc42

Cells were lysed using Laemmli's reducing sample buffer. The lysate was subjected to electrophoresis on a 7.5%-gradient polyacrylamide gel, and the proteins were transferred to a membrane (Immun-Blot PVDF; BIO-RAD, Hercules, CA, USA). The ABCA1 protein was detected using a rabbit anti-ABCA1 antibody (1:5000; Novus Biologicals, Littleton, CO, USA). Immunodetection was performed using a horseradish peroxidase-labeled anti-rabbit IgG antibody and western blotting detection reagents (Amersham ECL Prime; GE Healthcare, Little Chalfont, UK). The Cdc42 protein was analyzed in a similar manner using a rabbit anti-Cdc42 antibody (1:5000; EPITOMICS; Burlingame, CA, USA) and β-actin using an anti-actin rabbit antibody (1:1000; Santa Cruz Biotechnology, Inc, Santa Cruz, CA, USA). Photographs of the membranes were analyzed using Molecular Imager, ChemDoc XRS+ with image Lab Software (BIO-RAD). Each band was quantified and the relative ratio against β-actin was described.

### The influence of cAMP and PKA inhibitors on ApoA-I induced insuline secretion in MIN6 cells

Involvement of cAMP/PKA as downstream pathway of ABCA1/Cdc42 was examined. When ApoA-I (25 μg/ml) was added to the medium, 100 μmol/l SQ22536 (cAMP inhibitor) (22) or 100 μmol/l Rp-cAMPS [cAMP-dependent protein kinase inhibitor (23)] were simultaneously added to the medium. The effect of each inhibitor on insulin secretion was measured in the medium of MIN6 cells.

### Statistical analysis

Values are expressed as mean ± SD. Two groups were compared using the Mann–Whitney *U*-test. Differences among groups were determined using analysis of variance (ANOVA) with Bonferroni/Dunn *post-hoc* correction. Statistical analyses were performed using StatView 5.0 (SAS Institute; Cary, NC, USA). Values of *p* < 0.05 were considered to be statistically significant.

## Results

### Glucose- or ApoA-I-mediated cAMP levels in MIN6 cells

**Figure 1:**
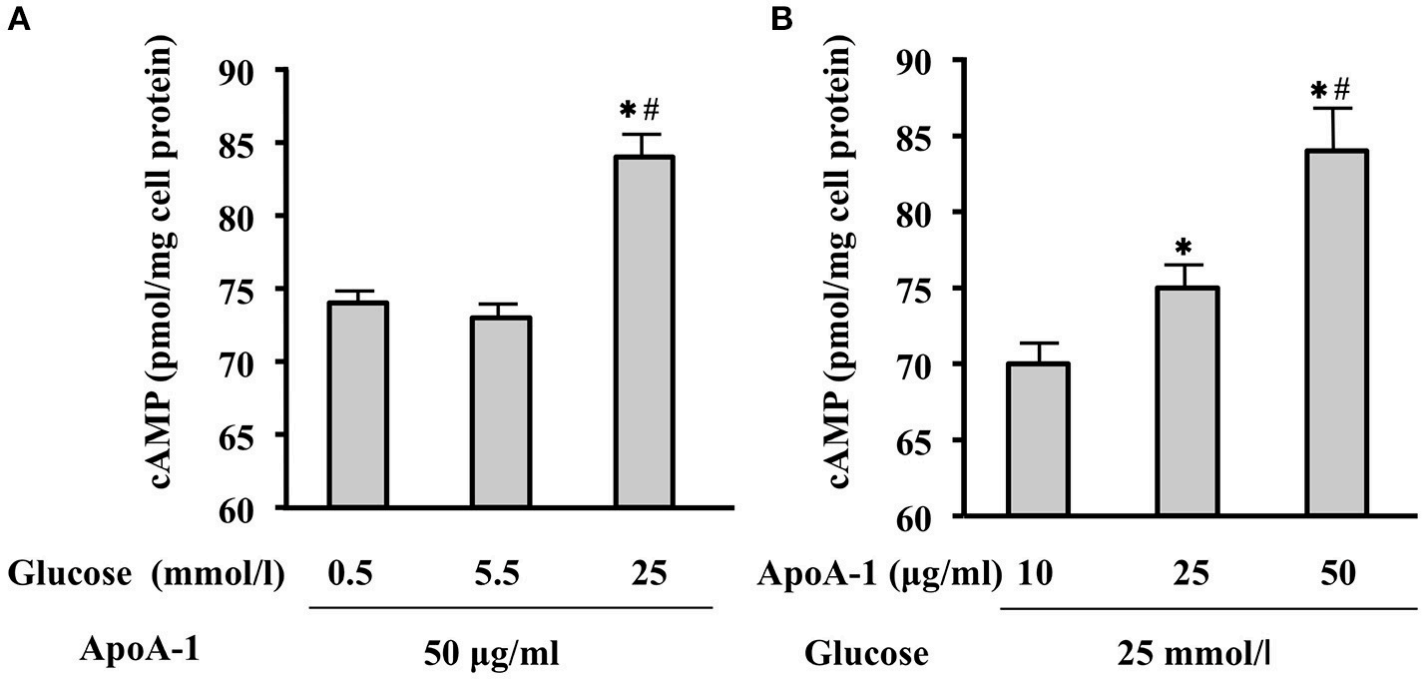
Glucose- or ApoA-I mediated changes in cAMP levels in MIN6 cells. When incubated with ApoA-I (50 ug/mL), cAMP levels in MIN6 cells cultured in the medium with 25 mmol/l glucose (high glucose) were significantly higher than those cultured in the medium with 0.5 mmol/L (low glucose) or 5.5 mmol/L (normal glucose) compared to those **(A)**. Incubation with ApoA-I (10, 25, and 50 ug/mL) increased cAMP levels from 70 to 84.5 pmol/mg cell protein in concentration-dependent manner in the medium with 25 mmol/l glucose **(B)**. Data were obtained from six independent experiments. **(A)** ^*^p < 0.05 vs. “0.5,” ^#^*p* < 0.05 vs. “5.5”; **(B)** ^*^p < 0.05 vs. “10”, ^#^*p* < 0.05 vs. “25”.

Incubated with ApoA-I (50 ug/ml), high glucose (25 mmol/l) increased MIN6 cAMP., however, glucose-mediated increase was not significant with low (0.5 mmol/l) or normal glucose level (5.5 mmol/l) (Figure 1A). Thereafter, cAMP production in MIN 6 cell by ApoA-I (10, 25, 50 ug /ml) in the presence of high glucose (25 mmol/l) were measured. Incubation with ApoA-I increased cAMP levels from 70 to 84.5 pmol/mg cell protein in concentration-dependent manner (Figure 1B). Then, we could confirm increase of cAMP by ApoA-I under high glucose.

### MIN6 cells secreted insulin in ApoA-I dependent manner in high glucose

**Figure 2:**
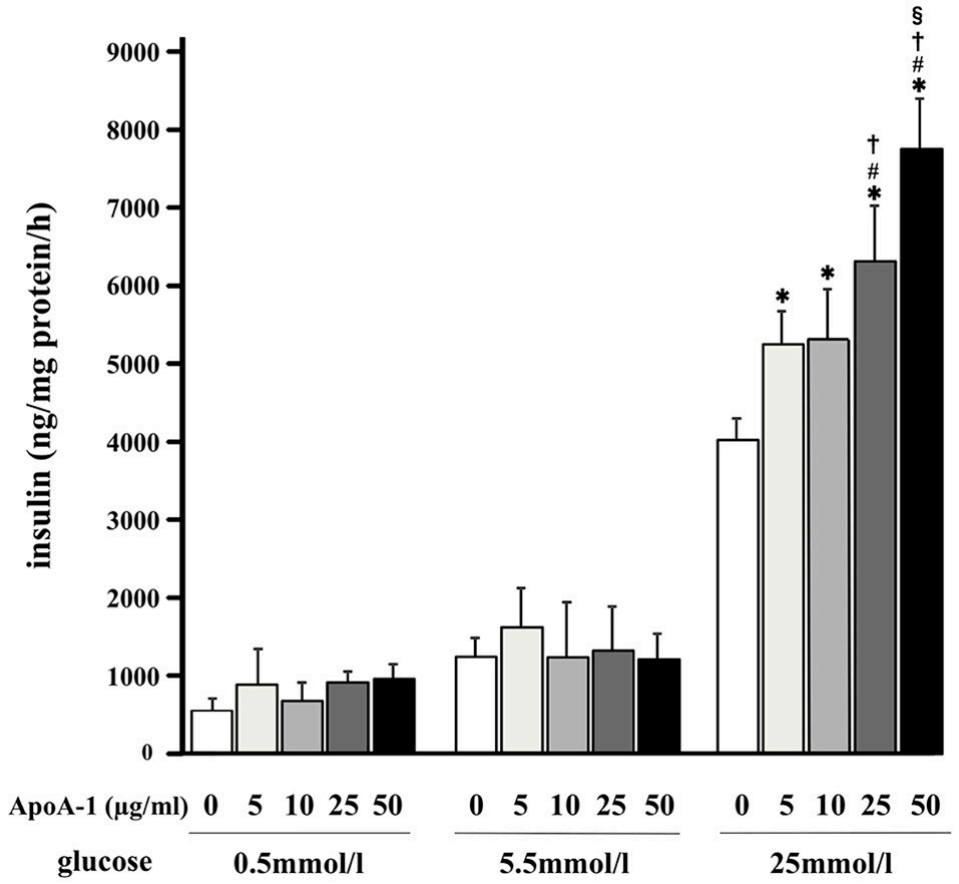
Glucose-stimulated insulin secretion and the insulinotropic action of ApoA-I on GSIS in MIN6 cells. MIN6 cells secreted insulin (ng insulin/mg protein/hour) in a glucose-dependent manner as shown in the column without ApoA-I. Addition of ApoA-I (5, 10, 25, 50 μg/ml) increased insulin secretion with dose dependent manner in 25 mmol/ml glucose. Data were obtained from eight independent experiments. ^*^p < 0.05 vs. “0,” ^#^*p* < 0.05 vs. “5,” *p* < 0.05 vs. “10,” ^§^*p* < 0.05 vs. “25”.

Without ApoA-I, MIN6 cells secreted insulin (ng insulin/mg protein/hour) in a glucose-dependent manner as shown in open columns in Figure 2. MIN6 cells secreted insulin in response to high glucose (25 mmol/ml) with ApoA-I dose dependent manner in Figure 2. However, the insulinotropic effect of ApoA-I was not apparent with low (0.5 mmol/l) or normal glucose (5.5 mmol/ml). These results were compatible with previous reports (13, 16).

### siRNA transfection of ABCA1 or Cdc42 inhibited ApoA-I induced insulin secretion with high glucose

**Figure 3:**
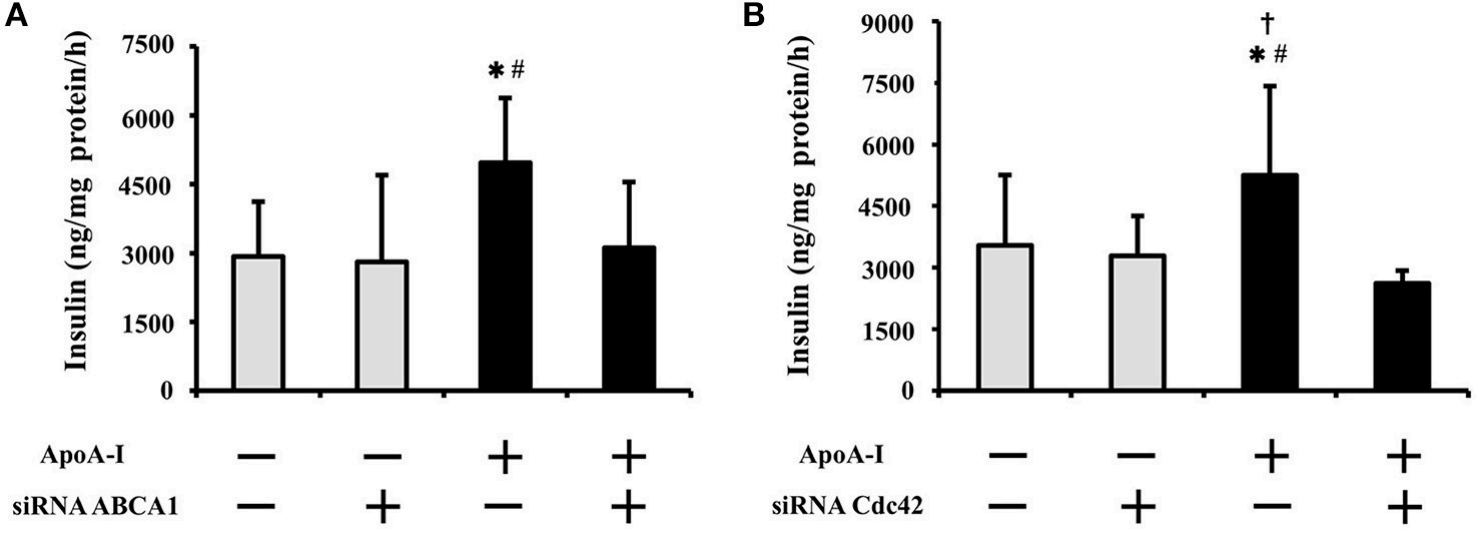
Effect of siRNA transfection of ABCA1 or Cdc42 on insulin secretion with high glucose (25 mmol/ml). ApoA-I significantly increased insulin secretion without siRNA (NC column) **(A,B)**. The insulinotropic effects of ApoA-I were significantly restrained by siRNA ABCA1 (*p* < 0.05; **A**) and Cdc42 (*p* < 0.05; **B**) transfection. In the condition without ApoA-I, the effect of siRNA of ABCA1 and Cdc42 on insulin secretion was not found **(A,B)**. Data were obtained from six independent experiments. ^*^p < 0.05 vs. “ApoA-1 – and siRNA–,” ^#^*p* < 0.05 vs. “ApoA-1 + and siRNA +,” *p* < 0.05 vs. “ApoA-1 – and siRNA +”.

To clarify the inhibitory effect of siRNA on insulin secretion, 50 μg/ml of ApoA-I was added into the medium with 25 mmol/l glucose (Figure 3). ApoA-I significantly increased insulin secretion without siRNA (NC column) (Figures 3A,B). The insulinotropic effects of ApoA-I (50 μg/ml) were significantly restrained by siRNA ABCA1 (*p* < 0.05; Figure 3A) and Cdc42 (*p* < 0.05; Figure 3B) transfection. In the condition without ApoA-I, the effect of siRNA of ABC1 and Cdc42 on insulin secretion was not found (Figures 3A,B).

Effects of siRNA for ABCA1 and Cdc42 were not apparent with low or normal glucose doses (0.5 and 5.5 mmol/l) (data not shown).

### Effect of siRNA transfection on ABCA1 or Cdc42 mRNA and protein expression with high glucose

**Figure 4:**
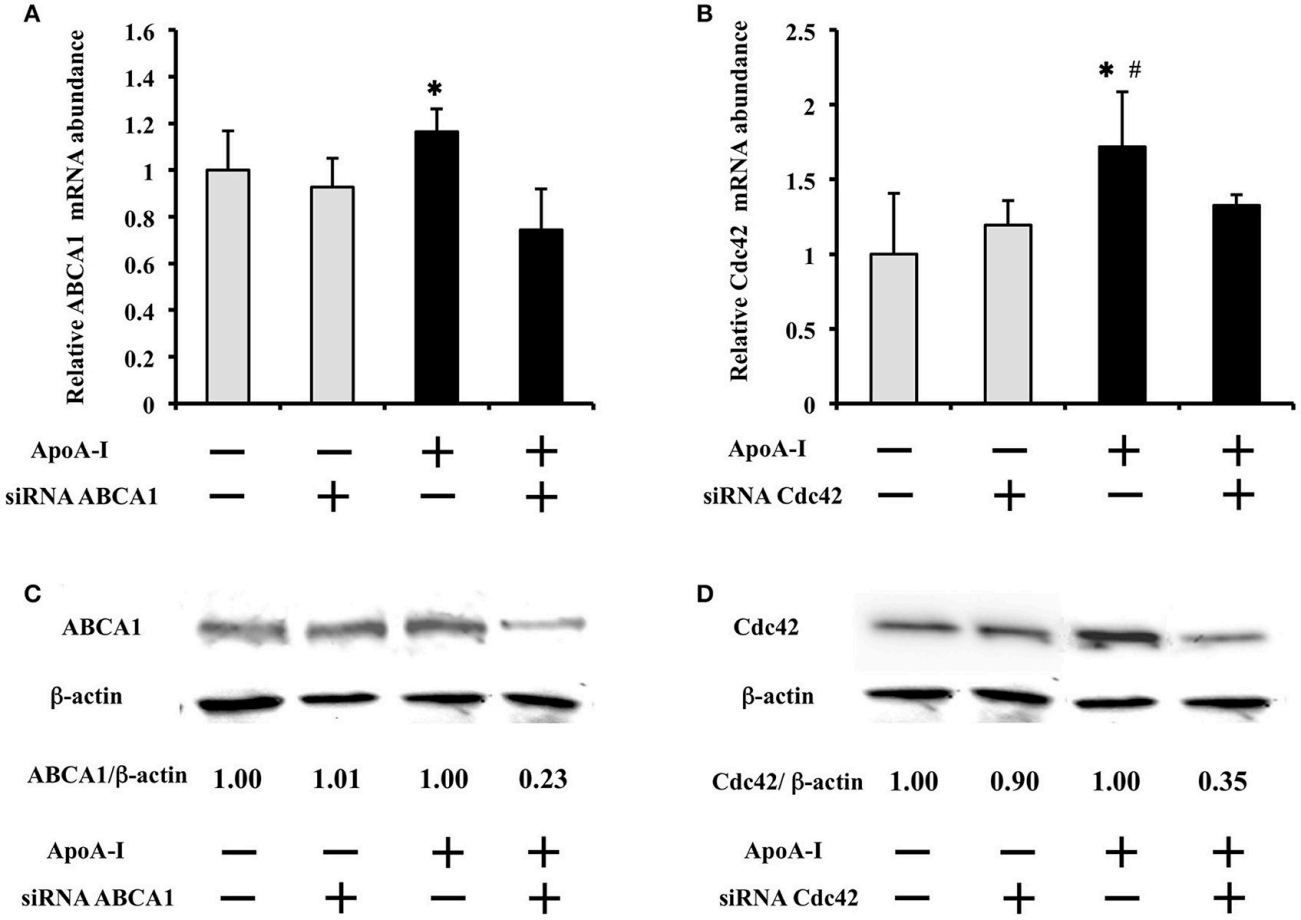
Effect of siRNA transfection on ABCA1 or Cdc42 mRNA and protein expression at high glucose condition (25 mmol/ml). ApoA-I showed tendency to increase ABCA1 mRNA (*p* = 0.10) **(A)** and significantly increased Cdc42 mRNA (*p* < 0.05) **(B)** Under the condition with ApoA-I, transfection of siRNA for ABCA1 decreased ABCA1 mRNA (*p* < 0.05) **(A)** and also protein expression **(C)** than seen with the negative control (NC) siRNA. Similarly, transfection of siRNA for Cdc42 showed a tendency (*p* = 0.06) to reduce Cdc42 mRNA **(B)** and decreased protein expression **(D)** compared with those with negative control siRNA. In the condition without ApoA-I, the effect of siRNA for ABCA1 and Cdc42 on their mRNA and protein was not found like insulin secretion (Figure 4). Data of relative mRNA abundance were obtained from six independent experiments, although the loading control was reused. ^*^p < 0.05 **(A)** ^*^p < 0.05 vs. “ApoA-1 + and siRNA +”; **(B)** ^*^p = 0.06 vs. “ApoA-1 + and siRNA +”, ^#^*p* < 0.05 vs. “ApoA-1 – and siRNA –”.

To clarify the effect of siRNA on mRNA and protein expression, 50 μg/ml of ApoA-I was added into the medium with 25 mmol/l glucose (Figure 4). ApoA-I showed tendency to increase ABCA1 mRNA (*p* = 0.10) and increased ABCA1 protein (Figure 4A), and Cdc42 mRNA (*p* < 0.05) and protein (Figure 4B) were significantly increased. Under the condition with ApoA-I, transfection of siRNA for ABCA1 decreased ABCA1 mRNA (*p* < 0.05) (Figure 4A) and protein expression (Figure 4C) than seen with the negative control siRNA. Transfection of siRNA for Cdc42 showed a tendency (*p* = 0.06) to reduce Cdc42 mRNA (Figure 4B) and decreased protein expression (Figure 4D) compared with those with negative control siRNA. In the condition without ApoA-I, the effect of siRNA of ABC1 and Cdc42 on their mRNA and protein was not found like insulin secretion (Figures 4A,B). These results indicate that ABCA1 and Cdc42 are involved in the insulin secretion mediated with ApoA-I.

### Both inhibitors of cAMP (SQ22536) and PKA (Rp-cAMPS) thoroughly inhibited the effects of ApoA-I on insulin secretion

**Figure 5:**
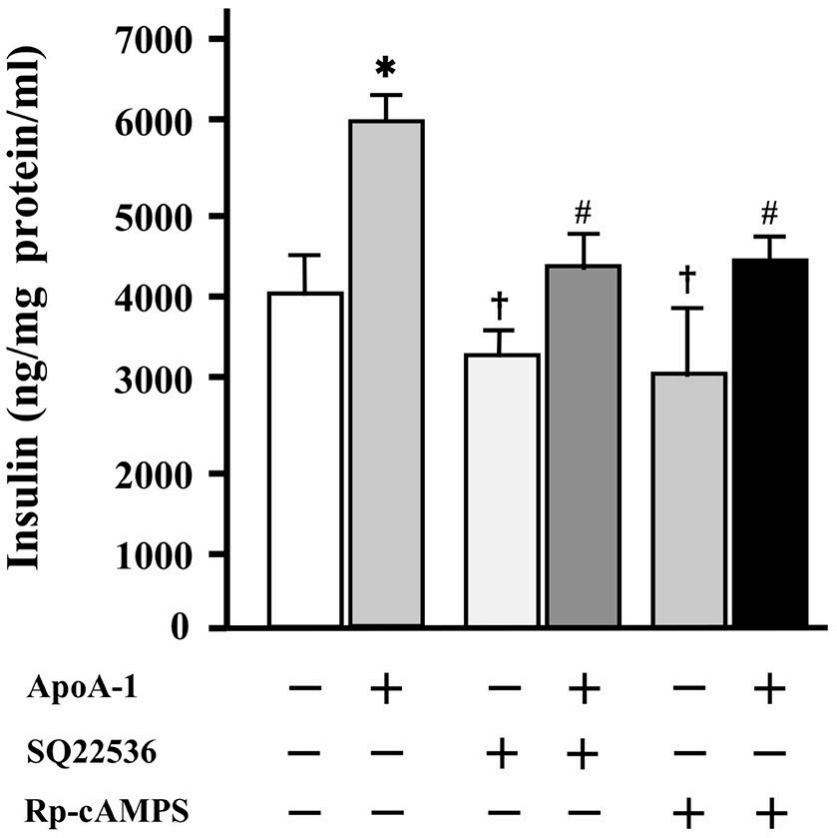
The influence of cAMP and PKA inhibitors on insulin secretion at high glucose codition (25 mmol/ml). Without ApoA-I, 100 μmol/l of SQ22536 or Rp-cAMPS accepted the tendency (*p* = 0.06) to decrease insulin secretion to glucose of 25 mmol/l, respectively. The insulinotropic effects of ApoA-I (25 μg/ml) were thoroughly inhibited with the addition of 100 μmol/l of SQ22536 or Rp-cAMPS. Data were obtained from six independent experiments. ^*^p < 0.05 vs. “ApoA-1 –, SQ22536 –, Rp-cAMPS –,” ^#^*p* < 0.05 vs. “ApoA-1 +, SQ22536 –, Rp-cAMPS –,” *p* = 0.06 vs. “ApoA-1 –, SQ22536 –, Rp-cAMPS –”.

Without ApoA-I, 100 μmol/l of SQ22536 or Rp-cAMPS accepted the tendency (*p* = 0.06) to decrease insulin secretion to glucose of 25 mmol/l, respectively. The insulinotropic effects of ApoA-I (25 μg/ml) were thoroughly inhibited with the addition of each inhibitor with 25 mmol/l glucose (Figure 5).

These results indicate that cAMP and PKA are involved in the insulin secretion mediated with ApoA-I.

## Discussion

It is not still clear wheather HDL-mediated signal activations are lipid-free apolipoprotein-related events, although a portion of the HDL apoproteins may dissociate to interact with cells (24). Actually, free ApoA-I, as well as native and recombinant HDL are reported to be able to stimulate insulin secretion by MIN6 cells and primary islets (13, 16), Then, we explored the possible mechanisms of insulinotropic actions of ApoA-I on glucose-stimulated insulin secretion (GSIS) in MIN6 cells. ApoA-I concentration of 25–50 ug/mL which we used for the experiment as other reports REFS is 1/20–1/60 concentration in the circulating blood ApoA-I level (50–100 mg/dl). Its concentration is not considered to have largely departed from the physiological range.

An increasing number of studies suggest that interaction with ApoA-I promotes the receptor-like properties of ABCA1 and activates signaling proteins such as protein kinase A (PKA), protein kinase C (PKC), Janus kinase 2 (JAK2), Rho family G protein Cdc42 and Ca2+; many other factors might also influence the interaction of ApoA-I with ABCA1 (15, 25). Therefore, HDL possesses many functional proteins when compared with other lipoproteins12. Actually, ApoA-I has been reported to activate small G protein Cdc42 signaling (26, 27), cAMP signaling (28–30), and downstream kinases through the ABCA1 transporter with the amounts of ApoA-I 1 to 50 μg/ml as our experiment (29). In fact, ApoA-I as cAMP-inducible lipoprotein (28), had been revealed to enhance Cdc42/cAMP/PKA pathway in THP-1 macrophages (18).

Furthermore, recent reports have demonstrated that ABCA1 play key roles in β-cell cholesterol homeostasis and insulin secretion (16). Mice with specific inactivation of ABCA1 in β cells showed markedly impaired glucose tolerance and defective insulin secretion (16). Furthermore, it has also been reported that Cdc42 signaling is essential for the second phase of insulin secretion in MIN6 cell (19).

We firstly demonstrated cAMP production in MIN6 cells mediated by high glucose and ApoA-I, and then, ApoA-I dose dependent insulin release in high glucose. Then, we clarified the possibility that ABCA1 and Cdc42 participate in ApoA-I induced insulin secretion pathway in MIN6 cells, showing that ApoA-I enhanced expression of mRNA and protein of Cdc42. As to ABCA1, ApoA-I enhanced ABCA1 protein, but mRNA. It has been controversial whether ApoA-I increases ABCA1 mRNA, because post-transcriptional regulation has been reported in ABCA1 responding to ApoA-I (30, 31). Then the insulinotrophic action of ApoA-I in 25 mmol/l glucose was restricted due to siRNA transfection against ABCA1 and Cdc42.

In our experiment, the suppression of insulin secretion and the inhibition of ABCA1 and Cdc42 mRNA and protein by their siRNA were not recognized in the condition where ApoA-I does not exist under high glucose, i.e., glucose-stimulated insulin secretion (GSIS) was not significantly inhibited without ApoA-I. There had been demonstrated that GSIS had deleted in isolated islets from ABCA1-KO mice (16) or depletion of Cdc42 by siRNA (19). We think it is a matter of how to choose siRNA. We had selected siRNA which effectively inhibit ApoA-I effect. Further, we revealed that the insulinotropic action by ApoA-I was restricted by incubation with SQ22536 (cAMP inhibitor) and Rp-cAMPS (cAMP-dependent protein kinase inhibitor). The insulin secretion by inhibitors for cAMP or PKA remained in declined tendency (p = 0.06) without ApoA-I. However, in the presence of ApoA-I (25–50 ug/ml), the suppressive effects of cAMP and PKA was remarkable. These findings support the possibility that ApoA-I secretes insulin through the cAMP/PKA pathway. ApoA-I induced secretion pattern was similar to those of active GLP-1 and the cAMP analog 8-Br-cAMP (data will be present elsewhere); these compounds are well-known to secrete insulin through amplifying pathway including the cAMP/PKA pathway (32).

ABCA1 was identified as the causative gene for Tangier disease, which is characterized by severe HDL deficiency and cholesterol accumulation in peripheral tissues and macrophages; this phenotype is also noted in knockout mice lacking ABCA1 (8). Fibroblasts in Tangier disease display decreased expression and activity of Cdc42, which controls cytoskeletal architecture and vesicular transport (33–35). Clinically, insulin secretion in patients with Tangier disease and the ABCA1 mutation is still under investigation. However, a case report of four patients with Tangier disease demonstrated that all patients had impaired glucose-stimulated insulin secretion (36). Another study has also demonstrated the association of ABCA1 gene polymorphisms with type 2 DM in a Japanese population (37).

However, this preliminary experiment has limited ability to clarify the extent to which the ABCA1-mediated signal pathway participates in insulin secretion under physiological conditions. As ApoA-I increases the intracellular cAMP, there is a possibility that PKA independent pathway i.e., cAMP/Epac2/Rap1 (38–40) may also be induced. Further investigations are necessary to identify the possibility of earlier insulin secretion via PKA independent pathway and the comparison with incretin in insulin secretion, which would elucidate the diversity of insulin secretion mechanisms by ApoA-I.

In conclusion, we have studied the mechanism underlying the insulinotropic action of ApoA-I in MIN6 cells. We directly showed the participation of ABCA1 and Cdc42 in insulin secretion, and demonstrated the possibility that ApoA-I enhances glucose-stimulated insulin release in high glucose, partially through the ABCA1/Cdc42/cAMP/PKA pathway (18). These findings will potentially reveal new therapeutic strategies for diabetes and its macroangiopathy.

## Author contributions

KM performed most of the experiments and researched data, contributed to discussion, wrote the article. MD contributed to discussion and reviewed and edited the article. NT researched data, contributed to discussion, wrote the article, and reviewed and edited the article.

### Conflict of interest statement

The authors declare that the research was conducted in the absence of any commercial or financial relationships that could be construed as a potential conflict of interest. The handling editor declared a shared affiliation, though no other collaboration, with the authors KM, MD at time of review.

## Abbreviations

ApoA-I: Apolipoprotein A-I
ApoB: Apolipoprotein B
ABCA1: ATP-binding cassette transporter A1
Cdc42: Cell division control protein 42 homolog
GAPDH: Glyceraldehyde 3-phosphate dehydrogenase
GLP-1: Glucagon-like peptide-1
GSIS: Glucose-stimulated insulin secretion
JAK2: Janus kinase 2
MIN6: Mouse insulinoma
PKA: Protein kinase A
PKC: Protein kinase C
RCT: Reverse cholesterol transport
Si: Small-interference
THP-1: Cells of a human monocytic leukemia cell line.
